# How Do Sleep Difficulties Interact With Anxiety, Depression and Health‐Related Quality of Life in Pulmonary Hypertension?

**DOI:** 10.1111/crj.70220

**Published:** 2026-07-31

**Authors:** Jemma L. Green, Vlad Costin, Iain Armstrong, Andrew R. Thompson, Gregg H. Rawlings

**Affiliations:** ^1^ School of Psychology University of Sheffield Sheffield UK; ^2^ School of Psychology University of Sussex Brighton UK; ^3^ Division of Clinical Medicine The University of Sheffield Sheffield UK; ^4^ Sheffield Pulmonary Vascular Disease Unit Royal Hallamshire Hospital, Sheffield Teaching Hospitals NHS Foundation Trust Sheffield UK; ^5^ South Wales Clinical Psychology Training Programme Cardiff and Vale University Health Board and Cardiff University Cardiff UK; ^6^ Clinical and Applied Psychology Unit University of Sheffield Sheffield UK

**Keywords:** anxiety, depression, distress, psychosocial, pulmonary arterial hypertension

## Abstract

Sleeping difficulties are prevalent among adults with pulmonary hypertension (PH), with most individuals studied to date reporting poor sleep. However, research investigating the impact of sleep on psychological distress and health‐related quality of life (HRQoL) in PH remains limited. This study aimed to identify the nature of common sleep difficulties in PH and the associated psychosocial impact using multiple self‐reported measures. A cross‐sectional survey was conducted with 111 adults with PH recruited from PH associations globally. Most identified as White (91%) and female (82%). Participants completed measures of overall sleep quality (Pittsburgh Sleep Quality Index), insomnia (Insomnia Severity Scale), daytime sleepiness (Epworth Sleepiness Scale), anxiety (Generalised Anxiety Disorder‐7), depression (Patient Health Questionnaire‐9) and HRQoL (emPHasis‐10). Regression analyses were used to inspect relationships between measures and other PH variables. A total of 79.8% of participants were identified as having poor sleep quality. The most prevalent sleep‐related difficulties were sleep disturbance and sleep latency issues. Insomnia and daytime sleepiness rates were also elevated (35% and 33.6%, respectively) and often co‐occurred. In addition, 40% and 29% of participants had clinical levels of depression and anxiety, respectively. All types of sleep difficulties were associated with lower HRQoL. A series of parallel mediation analyses suggested that this association was partially mediated by depressive symptoms. The findings indicate the need to routinely screen for both sleep difficulties and mood disturbance in this population. Further work is needed to understand whether existing brief sleep interventions are effective for use in PH, where multiple factors are influencing sleep quality.

## Graphical abstract

Our findings support that of others suggesting that most adults with pulmonary hypertension report difficulties with sleep, highlighting the importance of routine screening. Where sleep difficulties are identified, screening for co‐occurring depression may also be required given the strong association between the two. Future research is needed to explore how best to support sleep in this clinical population.

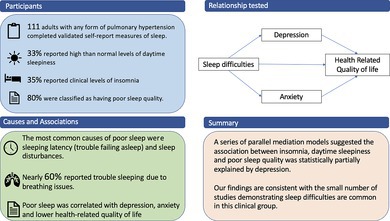

## 1 Introduction

Pulmonary hypertension (PH) refers to a group of serious conditions characterised by high blood pressure in the arteries connecting the heart to the lungs. This increased strain reduces cardiac efficiency and causes progressive damage, which can ultimately lead to heart failure if untreated. PH affects approximately 1% of the global population [1], with a relatively small proportion needing specialist care—in the United Kingdom, this is estimated to be 7–8000 people. Although treatments for PH are advancing, most forms of the disease remain life‐reducing and incurable [2].

PH is characterised by a range of debilitating symptoms, including severe breathlessness and fatigue [3]. Moreover, medical comorbidities are common in PH, including mental health difficulties such as anxiety and depression [4]. Although these conditions are distressing in themselves, there is evidence to suggest that they may interact with PH‐related symptoms [5]. For example, Rawlings et al. found that anxiety and depression statistically mediated the association between pain or fatigue and health‐related quality of life (HRQoL) in PH in a cross‐sectional sample [6]. This paper adds to the substantial evidence base documenting the negative impact of PH—and associated comorbidities—on HRQoL.

Although it has received limited attention, PH is frequently accompanied by sleep‐related difficulties, with data suggesting that most (> 50%) individuals interviewed to date are burdened. However, it is important to recognise that sleep problems are not a cardinal symptom of PH, unlike dyspnoea or fatigue. Matura et al. interviewed 191 patients with pulmonary arterial hypertension (PAH) living in the United States, revealing that 57% self‐reported problems with their sleep as measured using one item from the PAHSIS [7]—a 17‐item self‐report measure exploring how much PAH symptoms interfere with patients' lives. Although acknowledging that single‐item measures can be used to assess sleep quality [8], sleep has been viewed as a multifaceted construct, representing different influencing factors [9]. Therefore, using a single item to assess sleep may create a vague depiction, lacking detail of how sleep manifests specifically in individuals with PH and may be less helpful for identifying treatment targets. Indeed, Batal et al. assessed sleep quality in 40 patients with PH, also living in the United States, using a series of validated self‐reported measures of sleep, namely, the Pittsburgh Sleep Quality Index (PSQI) [10], Insomnia Severity Scale (ISI) [11] and Epworth Sleepiness Scale (ESS) [12]. Although 72.5% were categorised as ‘poor sleepers’, a more nuanced analysis revealed that 47.5% experienced some degree of insomnia, whereas 26% experienced excessive daytime sleepiness [13]. These rates are comparable with samples outside of the United States, as Sarzyńska and Jankowska‐Polańska administered the PSQI to 89 patients with PAH in Poland, reporting that 70% had poor sleep quality [14].

As documented in other respiratory‐related conditions, such as asthma [15], sleep‐related difficulties are likely to have a complex relationship with PH symptoms and associated comorbidities, given the overlapping or interacting symptoms. For example, depression, anxiety and sleep tend to co‐occur and influence one another: Poor sleep can impact mood, which can increase anxiety, resulting in pre‐sleep worry and physiological arousal, making it difficult to fall asleep. Low mood can cause the individual to sleep too much or too little, which also can disrupt the sleep–wake cycle. These negative cycles have a detrimental impact on HRQoL and are commonly addressed within interventions for sleep difficulties, including Cognitive Behavioural Therapy [16]. More specific to PH, shortness of breath and pain are closely linked to panic and anxiety, whereas fatigue can be correlated with depression [6]. Additionally, sleep‐related breathing disorders are common in PH, such as sleep apnoea and restless leg syndrome [17], which may feed into the proposed pathways.

Studies to date in PH have explored sleep and its impact using correlational or multiple regression analyses. Problems with sleep in PH have been associated with reduced HRQoL and physical exercise capacity [18] and increased depression, anxiety, dyspnoea and other PH symptoms [7, 13, 14, 19]. Although these methods have helped identify which symptoms have a predictive relationship, they fail to account for how sleep may impact HRQoL through multiple interconnected mediators, such as anxiety and depression. Therefore, in the current study, we examine the impact of different types of sleep difficulties in PH on HRQoL. We predict that sleep quality, insomnia and daytime sleepiness would be associated with HRQoL, and these relationships would be partly accounted for by anxiety and depression. In other words, poor sleep was expected to be associated with lower HRQoL both directly and indirectly through higher levels of anxiety and depression.

## 2 Materials and Methods

### 2.1 Design

A cross‐sectional design was utilised. The study was conducted in line with the Strengthening the Reporting of Observational Studies in Epidemiology (STROBE) guidelines [20] and preregistered on the Open Science Framework. At the point of preregistration, data collection had closed; however, no inspection of the data had been conducted. Ethical approval was obtained from the University of Sheffield, Psychology Department Ethics Committee (067112).

### 2.2 Participants

Convenience sampling was used as participants were recruited via a study advert promoted by PH Associations around the world, specifically PHA UK. To be eligible, participants were required to have a diagnosis of PH, which they were asked to self‐report as opposed to confirming through reviewing medical records, be aged 18 years or older and be able to complete online questionnaires in English without help. Data was collected between 12 and 21 May 2025.

### 2.3 Procedure

The study advert directed participants to a participant information sheet and consent form. After providing informed consent, they were asked to complete a series of outcome measures (see below). All study‐related documents were hosted by Qualtrics. Participants could withdraw their data up to 2 weeks after taking part. All participants were signposted to relevant services should they become distressed by the measures or require additional support.

### 2.4 Measures

#### 2.4.1 Demographic Information

Participants were asked to report their demographics including age, gender, ethnicity and nationality. PH‐related factors—type of PH, functional class and length of diagnosis—were also asked.

#### 2.4.2 Sleep

Sleep‐related difficulties were measured using three measures:

*Daytime sleepiness* was measured using the ESS [12]. This comprised eight items asking in what situations the participant is likely to fall asleep, that is, sitting and reading. Participants were asked to rate scenarios from 0 ‘would never doze’ to 3 ‘high chance of dozing’. Items were summed to create a total ESS score following the developer's instructions. A total score of 0–5 is indicative of lower normal daytime sleepiness, 6–10 higher normal daytime sleepiness, 11–12 mild excessive daytime sleepiness, 13–15 moderate excessive daytime sleepiness and 16–24 severe excessive daytime sleepiness. A score > 10 is suggested as the clinical cut‐off, indicating excessive daytime sleepiness. The ESS had good internal consistency (Cronbach's alpha = 0.86).

*Overall sleep quality* was measured using the PSQI. This 19‐item measure assesses seven domains of sleep: subjective sleep quality, sleep duration, sleep disturbance, sleep latency, daytime dysfunction due to sleepiness, sleep efficiency and use of sleeping medication. Responses range from 0 to 3, with a higher score indicating greater sleep disturbance over the last month. A total score was created following guidance from Buysse et al. [10] to create a global PSQI score, which could range from 0 to 21. As recommended, scores were grouped into ‘good sleep quality’ (a score ≤ 5) or ‘poor sleep quality’ (a score > 5). The PSQI also contains two open‐ended questions relating to self‐reported reasons for sleep disturbance and signs of restlessness, reported by a bed partner. This data does not contribute towards the global PSQI score calculation. Internal consistency was acceptable (Cronbach's alpha = 0.75).

*Insomnia* was measured using the ISI [11]. This is a seven‐item measure used to assess the severity and impact of insomnia over the last 2 weeks. Responses for individual items range from 0 (no problem) to 4 (very severe problem). Total scores can range from 0 to 28. As per the scoring instructions, items were summed to create a total ISI score and grouped into ‘no clinically significant insomnia’ (0–7), ‘subthreshold insomnia’ (8–14), ‘clinical insomnia (moderate severity)’ (15–21) and ‘clinical insomnia (severe)’ (22–28). A score ≥ 15 is used as a clinical cut‐off for detecting clinical insomnia. Internal consistency was good (Cronbach's alpha = 0.89).

As reported, the ESS, PSQI and ISI have previously been administered to people with PH. Permission to use these measures was granted by ePROVIDE.

#### 2.4.3 HRQoL

This was measured using the emPHasis‐10 [21], a 10‐item scale developed specifically for people with PH. Responses range from 0 to 5, with a higher score indicating a worse HRQoL. Item responses were summed to create a total emPHasis‐10 score, which could range from 0 to 50. Internal consistency was excellent (Cronbach's alpha = 0.90).

#### 2.4.4 Depression

Depression was measured using the Patient Health Questionnaire‐9 (PHQ‐9) [22]. Participants were asked to rate the frequency of nine symptoms of depression over the last 2 weeks. Responses can range from 0 to 3, with a higher score indicating more severe depression. Items were summed to create a total score, which could range from 0 to 27. Scores were grouped into the following categories: ‘no depression’ (0–4), ‘mild’ (5–9), ‘moderate’ (10–14), ‘moderately severe’ (15–19) and ‘severe’ (20–27). A score ≥ 10 indicates a clinical case of depression. Internal consistency of the measure was excellent (Cronbach's alpha = 0.90).

#### 2.4.5 Anxiety

Anxiety was measured using the Generalised Anxiety Disorder‐7 (GAD‐7) [23]. This is a seven‐item scale measuring the frequency of anxiety symptoms over the last 2 weeks. Responses range from 0 to 3, with higher scores indicating more severe anxiety. Items were summed to create a total GAD‐7 score, which could range from 0 to 21. Scores could indicate ‘minimal anxiety’ (0–4), ‘mild anxiety’ (5–9), ‘moderate anxiety’ (10–14) and ‘severe anxiety’ (≥ 15). A score ≥ 8 suggests clinical levels of anxiety. Internal consistency was excellent (Cronbach's alpha = 0.93).

### 2.5 Data Analysis

Data cleaning and creation of total scores for outcome measures (ESS, ISI, PSQI, emPHasis‐10, GAD‐7 and PHQ‐9) were completed using Microsoft Excel. The midpoint was used in PSQI scoring when a time range was provided, in accordance with guidance from Buysse et al. Descriptive statistics (mean, standard deviation [SD], *n* and percentages) were used to analyse the frequency of difficulties. All other descriptive and inferential analyses were performed in IBM SPSS Statistics (Version 29) or R v4.5.0.

To investigate the relationship between continuous variables, a correlation analysis was performed. Age and length of diagnosis were not normally distributed, and scatter plots demonstrated a violation of the assumption of linearity for multiple continuous variables. Therefore, a non‐parametric Spearman's rank analysis was deemed appropriate.

To investigate if levels of daytime sleepiness, sleep quality, insomnia, HRQoL, anxiety or depression differed significantly between groups for gender, a series of independent *t*‐tests were conducted, with groups ‘other’ and ‘prefer not to say’ treated as missing due to small sample sizes. Due to the unequal group sizes, Welch's *t*‐tests were applied to not assume equal variance.

To examine differences in outcome measures between types of PH, a series of one‐way ANOVAs were conducted. Due to small sample sizes (*n* < 10) in several groups, these levels were excluded from analysis. A series of one‐way ANOVAs were conducted with the remaining groups: ‘idiopathic PAH’, ‘PH caused by heart problems’ and ‘chronic thromboembolic PH (CTEPH)’. Due to insufficient group sizes (*n* < 5 in several categories), a series of one‐way ANOVAs could not be conducted to examine the effect of ethnicity or country of residence on outcome measures.

To investigate the unique contribution of different types of sleep difficulties, anxiety and depression predicting HRQoL, we specified three linear models with multiple predictors. All models had HRQoL as the outcome and depression and anxiety as predictors, but each model used a different measure of sleep difficulty: daytime sleepiness (ESS), overall sleep quality (PSQI) or insomnia (ISI). After inspecting residual plots for signs of violated assumptions, we tested whether parameter estimates, confidence intervals and *p*‐values had been biased using the lmRob function from the robust package (v0.7‐5; [24]). Where non‐robust estimates were substantially different from their robust versions—for instance, by suggesting different patterns of significance—the more conservative robust versions were reported. To test the relative importance of predictors in each linear model while circumventing issues of multicollinearity, we ran dominance analyses using the dominanceanalysis package (v2.1.0; [25]). We then estimated how often the observed dominance pattern would be reproduced across 5000 bootstrapped samples [26]. Following from these linear models, we explored mechanistic explanations of the effect of sleep on HRQoL. Three mediation models—one for each sleep difficulty variable—were specified using the lavaan package (v0.6.17; [27]). All models included depression and anxiety as parallel mediators of sleep difficulty and HRQoL. Mediation models were specified using bootstrapped standard errors (1000 samples).

Listwise deletion was used for missing data within correlational and regression analyses. For mediation models, missing values were handled using full‐information maximum likelihood. Pairwise deletion of missing data within a correlational analysis was inspected for differences. The quantity of statistically significant correlations remained stable; however, the weak correlation between age and years of diagnosis was no longer significant (*r*_s_(109) = −0.16, *p* = 0.089). Due to the sample size in listwise deletion remaining higher than the minimum sample size required (*N* = 77) and the results of the Spearman's rank analysis impacting the rationale for further analysis, listwise deletion was appropriate. PH functional class was treated as ordinal data; however, due to many participants responding ‘not sure’, this data was treated as missing and not involved in the listwise deletion Spearman's rank correlation analysis. See Supporting Information for the Spearman's rank correlation matrix involving pairwise deletion of missing data and PH functional class.

To analyse the two open‐ended questions in the PSQI, an inductive approach to content analysis was used. After familiarisation with the data, the lead author identified key themes to code responses. Frequency and percentage of prevalence of themes were calculated. Generative AI was used to support improvements in grammar, spelling and argument clarity.

### 2.6 Patient and Public Involvement

The design and procedure of the study were developed in association with PHA UK and medical experts in PH.

## 3 Results

### 3.1 Demographics

In total, 114 participants provided informed consent; however, three did not complete an outcome measure and so were excluded. Most participants were female and from the United Kingdom and self‐reported their ethnicity as White. The mean age of individuals was 58.3 (SD = 14.6, range 19–90). Participants had been living with PH on average 8.1 years (SD = 8.2). Idiopathic PH was the most reported type of PH (38.7%), followed by CTEPH (24.3%). Over half of the sample did not know their functional class (51.8%); of those who did, a functional class of III was most prevalent (see Table 1).

**TABLE 1:** Participant characteristics.

	Mean (SD)	*N* (%)
Age	58.28 (14.62)	
Gender		
Female		91 (82.0%)
Male		18 (16.2%)
Other		1 (0.9%)
Prefer not to say		1 (0.9%)
Ethnicity		
White		101 (91.0%)
Asian or Asian British		5 (4.5%)
Mixed or multiple ethnic groups		1 (0.9%)
Other		4 (3.6%)
Country of residence		
United Kingdom		105 (94.6%)
Ireland		3 (2.7%)
New Zealand		1 (0.9%)
United States		1 (0.9%)
Serbia		1 (0.9%)
Length of diagnosis (years)	8.08 (8.22)	
Type of PH		
Idiopathic PAH		43 (38.7%)
Genetic or hereditary causes of PAH		6 (5.4%)
PAH caused by connective tissue disorder		6 (5.4%)
PAH but unsure which type		4 (3.6%)
Caused by heart problems (e.g., heart valve disease)		12 (10.8%)
Caused by lung problems (e.g., COPD)		6 (5.4%)
CTEPH		27 (24.3%)
Another cause of PH		3 (2.7%)
Unsure		4 (3.6%)
WHO functional class (missing data *N* = 1)		
Class I		7 (6.4%)
Class II		19 (17.3%)
Class III		26 (23.6%)
Class IV		1 (0.9%)
Unsure		57 (51.8%)

Abbreviations: COPD = chronic obstructive pulmonary disease, CTEPH = chronic thromboembolic pulmonary hypertension, PAH = pulmonary arterial hypertension, SD = standard deviation.

### 3.2 Scores on Psychological Distress, Sleep and HRQoL

Scores on the ESS indicated that 33.6% experienced higher than normal daytime sleepiness. The most common situation to doze off in was watching TV or lying down to rest, whereas the least common was sitting and talking to someone or in a car while stopped for traffic.

ISI scores suggested that 35% had clinical levels of insomnia. Additionally, 74.8% (*n* = 77/103) thought the impact that their poor sleep was having on their quality of life was noticeable to others. Eighty‐four per cent (*n* = 86/103) reported that their sleep interfered with their daily functioning (e.g., daytime fatigue, ability to function at work/daily chores, concentration, memory and mood). Of the participants, 72.8% (*n* = 75/103) were worried about their sleep. Overall, 62.2% (*n* = 23/37) of those with clinical levels of daytime sleepiness also reported clinical levels of insomnia, and 63.9% (*n* = 23/36) of those with insomnia also had clinical levels of daytime sleepiness.

Overall, 79.8% of participants were classified as having ‘poor sleep quality’ according to the PSQI. In comparison, when asked to rate their overall sleep quality, 45.4% (*n* = 49/108) reported it as ‘very’ or ‘fairly bad’. A review of the seven domains of the PSQI indicated that sleep disturbance and sleep latency had the highest mean score, suggesting that participants struggled the most with taking a long time to fall asleep and having frequent disruptions to their sleep. The PSQI asks about trouble sleeping because of breathing issues, with 42.6% (*n* = 46/108) not having this problem in the last month. Use of sleeping medication had the lowest mean score, as 77.6% (*n* = 83/107) had not taken medication to help sleep in the past month. Participants slept for an average of 6.5 h per night (SD = 1.6).

Most participants reported minimal or mild levels of anxiety (77.4%) and depression (58.6%). Moreover, 29% exceeded the clinical cut‐off for anxiety and 41.5% for depression. The mean HRQoL score was 27.30. See Table 2 for more information.

**TABLE 2:** Results from outcome measures of functioning.

	Mean score (SD)	*N* (%)
Daytime sleepiness (ESS)	8.25 (5.11)	
Lower normal daytime sleepiness		40 (36.4%)
Higher normal daytime sleepiness		33 (30.0%)
Mild excessive daytime sleepiness		10 (9.1%)
Moderate excessive daytime sleepiness		18 (16.4%)
Severe excessive daytime sleepiness		9 (8.2%)
Above the clinical cut‐off		37 (33.6%)
Sleep quality (PSQI)	9.62 (4.23)	
Good sleep quality		21 (20.2%)
Poor sleep quality		83 (79.8%)
Subjective sleep quality (possible score 0–3)	1.51 (0.79)	
Sleep duration (possible score 0–3)	0.96 (1.08)	
Sleep disturbance (possible score 0–3)	2 (0.63)	
Sleep latency (possible score 0–3)	2 (1.03)	
Sleep dysfunction (possible score 0–3)	1 (0.78)	
Sleep efficiency (possible score 0–3)	1.62 (1.16)	
Use of sleeping medication (possible score 0–3)	0.56 (1.09)	
Insomnia (ISI)	11.89 (6.44)	
No clinically significant insomnia		30 (29.1%)
Subthreshold insomnia		37 (35.9%)
Moderate clinical insomnia		29 (28.2%)
Severe clinical insomnia		7 (6.8%)
Above the clinical cut‐off		36 (35%)
HRQoL (emPHasis‐10)	27.30 (10.57)	
Depression (PHQ‐9)	9.33 (6.64)	
No depression		26 (27.7%)
Mild depression		29 (30.9%)
Moderate depression		19 (20.2%)
Moderately severe depression		12 (12.8%)
Severe depression		8 (8.5%)
Clinical level of depression		39 (41.5%)
Anxiety (GAD‐7)	6.08 (5.65)	
Minimal anxiety		48 (51.6%)
Mild anxiety		24 (25.8%)
Moderate anxiety		11 (11.8%)
Severe anxiety		10 (10.8%)
Clinical level of anxiety		27 (29%)

Abbreviation: SD = standard deviation.

### 3.3 Relationship Between Demographics, Psychological Distress, Sleep and HRQoL

All sleep‐related measures were significantly correlated with HRQoL. More specifically, daytime sleepiness and insomnia were moderately positively correlated with HRQoL, whereas overall sleep quality was weakly positively correlated. Depression and anxiety were strongly and moderately positively correlated with HRQoL and strongly positively correlated with each other. Insomnia was moderately positively correlated with overall sleep quality and daytime sleepiness, whereas the relationship between overall sleep quality and daytime sleepiness was statistically significant but weak. Duration since PH diagnosis was not significantly correlated with any outcome measure. Age was weakly correlated with daytime sleepiness—with older participants less likely to sleep during the day (see Table 3). After correcting for multiple comparisons, total scores on none of the measures differed significantly between groups based on gender or type of PH.

**TABLE 3:** Spearman's rank correlations for continuous variables and outcome measures.

Variable	1	2	3	4	5	6	7	8
1 emPHasis‐10 (HRQoL)	—							
2 ESS (daytime sleepiness)	0.51**	—						
3 PSQI (overall sleep quality)	0.29**	0.28*	—					
4 ISI (insomnia)	0.51**	0.45**	0.69**	—				
5 PHQ‐9 (depression)	0.78**	0.50**	0.45**	0.62**	—			
6 GAD‐7 (anxiety)	0.66**	0.45**	0.32**	0.50**	0.76**	—		
7 Age	−0.02	−0.28*	0.04	< 0.01	−0.06	−0.28*	—	
8 Duration since PH diagnosis	−0.14	0.09	−0.03	0.03	−0.07	−0.05	−0.28*	—

*Note: N* = 77.

Abbreviation: HRQoL = health‐related quality of life.

* *p* < 0.05.

** *p* < 0.01.

### 3.4 Impact of Sleep Difficulties, Anxiety and Depression on HRQoL

As shown in Table 4, in the three models where sleep difficulty variables, depression and anxiety were entered as simultaneous predictors of HRQoL, only depression was significant (*p*s < 0.001). In follow‐up dominance analyses shown in Table 5, across models, depression completely dominated sleep difficulty variables (reproduced in 92.9%–99.9% of bootstrapped samples) and anxiety (reproduced in 99.4%–99.9% of bootstrapped samples).

**TABLE 4:** Linear models predicting HRQoL from sleep difficulties, depression and anxiety.

Variable	*b* [95% CI]	*β*	*t*	*p*
	Model with daytime sleepiness (ESS)^a^			
ESS (daytime sleepiness)	0.15 [−0.30, 0.60]	0.07	0.67	0.503
**PHQ‐9 (depression)**	**0.86 [0.37, 1.35]**	0.**54**	**3.47**	**< 0.001**
GAD‐7 (anxiety)	0.20 [−0.33, 0.72]	0.11	0.75	0.457
	Model with overall sleep quality (PSQI)			
PSQI (overall sleep quality)	0.02 [−0.42, 0.47]	< 0.01	0.10	0.919
**PHQ‐9 (depression)**	**1.05 [0.62, 1.48]**	0.**67**	**4.88**	**< 0.001**
GAD‐7 (anxiety)	0.16 [−0.31, 0.63]	0.09	0.67	0.505
	Model with insomnia (ISI)			
ISI (insomnia)	0.13 [−0.21, 0.48]	0.07	0.76	0.451
**PHQ‐9 (depression)**	**0.99 [0.56, 1.42]**	0.**63**	**4.57**	**< 0.001**
GAD‐7 (anxiety)	0.15 [−0.30, 0.61]	0.08	0.67	0.507

*Note:* Significant relationships are in bold.

^a^ Robust model estimates.

**TABLE 5:** Bootstrapped dominance analysis across 5000 samples: sleep difficulties, depression and anxiety as predictors of HRQoL.

*i*	*j*	*Dij*	*Pij*	*Pji*	*Pnoij*
		Model with daytime sleepiness (ESS)			
Sleep difficulties	Depression	0	0.001	0.929	0.070
Sleep difficulties	Anxiety	0.5	0.101	0.141	0.757
Depression	Anxiety	1.0	0.994	< 0.001	0.005
		Model with overall sleep quality (PSQI)			
Sleep difficulties	Depression	0	0.000	0.999	0.001
Sleep difficulties	Anxiety	0	0.004	0.510	0.485
Depression	Anxiety	1	0.999	0.000	0.001
		Model with insomnia (ISI)			
Sleep difficulties	Depression	0	< 0.001	0.995	0.004
Sleep difficulties	Anxiety	0.5	0.066	0.425	0.510
Depression	Anxiety	1	0.996	< 0.001	0.004

*Note: Dij* = pattern of dominance in the current sample (1 = *i* dominates *j*, 0 = *j* dominates *i*, 0.5 = neither predictor dominates the other); *Pij* = proportion of samples where *i* dominates *j*; *Pji* = proportion of samples where *j* dominates *i*; *Pnoij* = proportion of samples with undetermined dominance between *i* and *j*.

**FIGURE 1:**
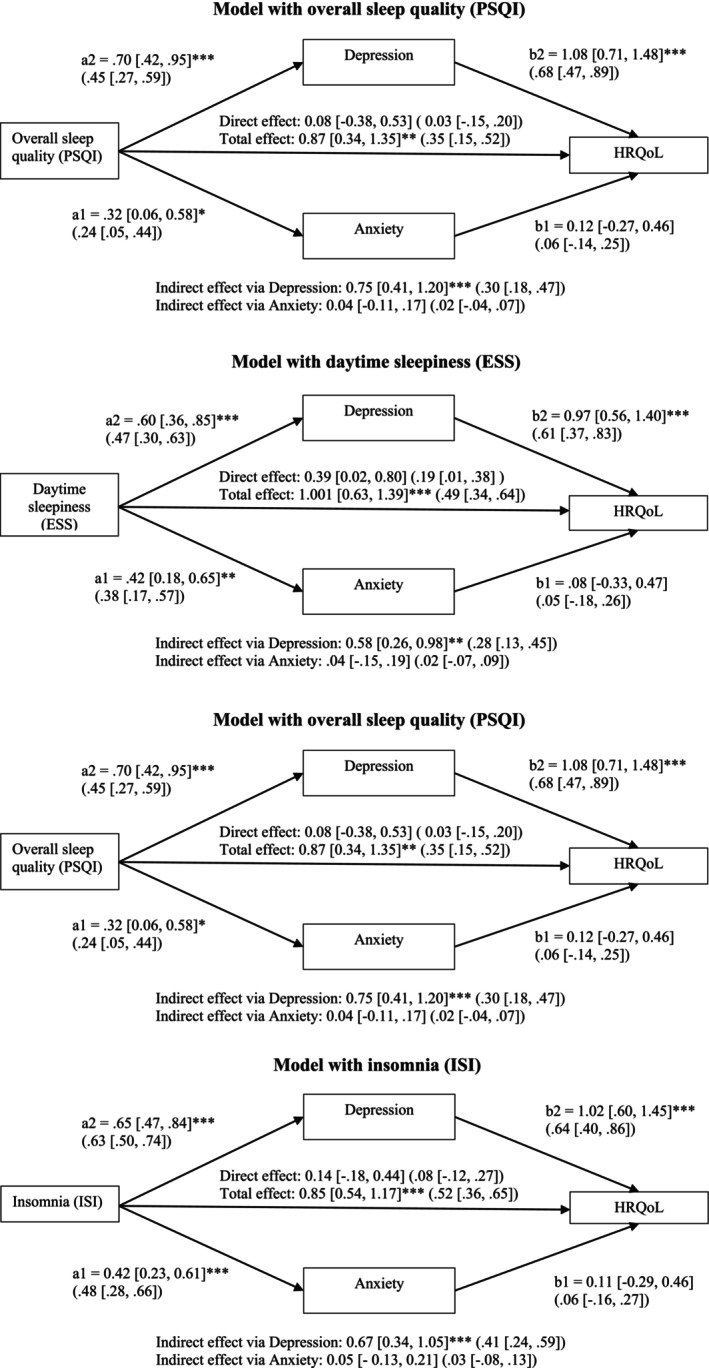
Parallel mediation models showing unstandardised estimates with bootstrapped 95% confidence intervals (and standardised estimates in brackets) for sleep difficulties on HRQoL through anxiety or depression. *p < 0.05, **p < 0.01, ***p < 0.001.

Parallel mediation analyses were performed to investigate whether depression and anxiety statistically mediated the association between sleep difficulties and HRQoL. As shown in Figure 1, analyses indicated that the association between the three types of sleep difficulties and HRQoL was statistically partially explained by depression: daytime sleepiness (ESS), *b =* 0.58, 95% CI [0.26, 0.98], *β* = 0.28, *p* = 0.001; overall sleep quality (PSQI), *b =* 0.75, 95% CI [0.41, 1.20], *β* = 0.30, *p* < 0.001; and insomnia (ISI), *b =* 0.67, 95% CI [0.34, 1.05], *β* = 0.41, *p* < 0.001. Once accounting for the indirect effect through depression, sleep difficulties no longer had a significant direct effect on HRQoL and indirect effects through anxiety were non‐significant (*p*s > 0.05).

### 3.5 Qualitative Analysis

Forty‐one participants provided reasons for their sleep difficulties in the PSQI. The most reported reasons were related to additional health conditions, specifically arthritis and Crohn's disease (24.3%). The next most common reason was related to anxiety, as one person explained: ‘Anxiety that if I go to sleep, I won't wake up.’ This was followed by physical discomfort and struggling to get to sleep (each 19.5%). Some aspects of physical discomfort appeared to relate specifically to PH, for example, breathlessness, but also encompassed general body pain and feeling uncomfortable—30.4% blamed their bed partner for tossing and turning. Difficulties with continuous positive airway pressure (CPAP) machines, used to treat sleep apnoea, were specifically reported: ‘My CPAP mask leaks at times causing restlessness.’ See Supporting Information for more information.

## 4 Discussion

This study aimed to better understand how sleep difficulties impact people with PH. The findings are consistent with previous research indicating that most of the sample (79.8%) were identified as poorer sleepers, as evaluated using a validated measure. This is higher than the estimated prevalence of 36% reported in the general population using the same measure [28]. Worryingly, only 45.4% of our sample self‐appraised their sleep as ‘bad’ when asked to directly rate their perceived quality of sleep using a single item in the PSQI, suggesting a possible lack of awareness. Findings support the practice of routine screening of sleep problems in PH to help identify those who need support.

Sleep difficulties were not fully explained by daytime sleepiness, which was experienced by 33.6% of the sample, or insomnia, which affected 35%. These rates can be compared to the general population, with evidence suggesting that 22.1% experience excessive daytime sleepiness [29] and 7.5% experience clinical insomnia using the same measures [30]. On average, participants slept for 6.5 h, which is similar to that reported by other samples with PH. For example, Tiede et al. [18] found that people with IPAH or CTEPH slept on average 7.1 and 7 h, respectively, which was comparable to healthy controls and the general population. Instead, findings from the current study suggest that sleep difficulties may be more related to falling asleep and being disturbed, rather than the duration of sleep. The majority reported that breathing difficulties impacted their sleep, which is consistent with other studies examining the relationship between dyspnoea and sleep quality in PH. Qualitative analyses in the current study help provide additional insight into potential causes including anxiety, consequences of other health conditions and disruption caused by a bed partner. However, no one cause was described by most participants, suggesting that interventions addressing poor sleep in PH may need to be individually tailored.

As expected, worse HRQoL was significantly correlated with greater levels of insomnia, daytime sleepiness, poor sleep, depression and anxiety. Indeed, most recognised that their sleep was interfering with their daily functioning (84%) and that it was noticeable to others (74.8%). A series of dominance analyses allowed us to understand which outcome was the best statistical predictor of HRQoL. When each sleep difficulty was assessed alongside anxiety and depression, depression emerged as the dominant predictor across all models. Additionally, parallel mediation analyses indicated that the association between insomnia, daytime sleepiness and poor sleep quality with HRQoL was statistically accounted for by depression. As such, our hypothesis was only partially accepted. The co‐occurrence of depression and poor sleep has previously been reported in samples with PH [13]. The results suggest that interventions aiming to improve HRQoL in PH may benefit from assessing for difficulties with depression and sleep problems.

Unfortunately, as far as we are aware, there are no published trials specifically targeting sleep in PH. Although sleep has been assessed in a range of treatment studies for PH, for example, when explored as part of an outcome measure such as the PAHSIS or PHQ [31], authors tend not to publish results on an individual item. Moreover, as previously discussed, there are issues of assessing sleep using a single‐item measure. However, due to the overlap between symptoms characteristic of depression and sleep problems, the results should be interpreted with caution. Notwithstanding the finding that outcome measures for depression and sleep were only moderately correlated, the PHQ‐9 asks about difficulties with sleeping too much or too little, which could account for some of the high variance.

Anxiety was statistically dominated by depression when in the same model and did not emerge as a significant mediator of the effect of sleep on HRQoL. Therefore, our hypothesis that anxiety would explain the relationship between the outcomes is rejected. However, the findings contrast with qualitative accounts as some participants self‐reported anxiety as a cause of their sleep problem and described high levels of sleep latency, which could be associated with rumination impacting their ability to fall asleep. Although this cognitive symptom is measured by the GAD‐7 (e.g., worrying too much and not being able to stop or control worrying), it is possible that the effect is diluted by responses to the other anxiety‐related items that may be less relevant to sleep. Findings may suggest an alternative path to the one we tested in which anxiety is associated with sleep difficulties, which in turn contributes to lower HRQoL. Given the importance of anxiety in treatments for sleep problems and participants' explanations, further research is required to better understand the role of anxiety when addressing sleep in PH.

We must recognise the high levels of anxiety and depression observed in a community sample of people with PH. The high prevalence of mood disorders is now well documented in PH. Moreover, our findings are consistent with previous research suggesting that anxiety and depression are not correlated with demographics in PH [4]. Although sleep quality and insomnia were not related to age, gender or type of PH, daytime sleepiness was related to age. Given that this relationship was weak, patients should be screened for sleep difficulties regardless of their demographics.

The study shares similar issues in terms of generalisability to other studies in the area as the sample was predominantly female [13, 14, 19]. Although this may be related to the female predominance in PH, we recognise that our results are largely reflective of sleep difficulties in women with PH. Furthermore, this may have contributed to inflated rates of depression in our sample than might be expected in studies with a greater representation of males, given that the condition is suggested to be more common among females [32]. Finally, the study sample was predominantly UK based (94.6%) and identified as White (91%). Although restricted through the convenience sampling design, it is therefore a limitation that the results are potentially unrepresentative of the experiences of all adults who are living with PH.

Unfortunately, due to insufficient sample sizes in categorical variable groups, the impact of ethnicity and country of residence on outcome measures could not be analysed. Similarly, due to the amount of missing data, we were unable to explore the relationship between functional class and outcome measures. Other research has shown that this may be an important factor as those in a higher class have reported greater impaired sleep quality [19]. It is not the first time we have found a lack of knowledge of functional class in a predominantly UK‐based sample [6]. The use of measures, such as the WHO functional class system, may be an effective intervention promoting self‐monitoring, increased awareness and feedback. It is possible that the usefulness of the WHO functional class as a measure of disease severity could be better utilised.

Recognising that participants discussed the impact of other health conditions on sleep quality, it is a limitation that we were unable to explore this further as details on comorbid diagnoses were not systematically captured, nor was BMI. For example, in Batal et al., 30% of the sample presented with symptoms consistent with restless leg syndrome [13]. This reflects the constraints of conducting research in a community sample, where detailed clinical data collection is often limited by considerations of participant burden and feasibility. In clinical studies, such information can more readily be obtained via medical records and routine assessments.

Our study shares a limitation with most trials in this area, which have examined sleep via self‐reported measures that ask about sleep retrospectively, rather than objective measures or ecological momentary assessment, which capture sleep prospectively. Although self‐reported sleep provides valuable subjective insight, these data can be supplemented with findings from objective measures of sleep [33], which may have additional implications for practice. Importantly, this is not to suggest that one method is superior; rather, different approaches capture distinct aspects of sleep and, when combined, provide a more comprehensive understanding. For example, self‐reported sleep tends to be moderately related to data obtained from objective methods, suggesting that people can over‐ or under‐estimate different aspects of their sleep, for example, duration and efficiency. These differences may vary depending on the method of self‐report and individual factors such as diagnosed sleep disorders [34]. In contrast, objective measures can provide detail on sleep architecture and nocturnal activity that cannot be captured via self‐report. Furthermore, although sleeping medication was, to some extent, measured through a PSQI domain, PH‐specific medication and treatments were not examined. Recognising sleep within the wider clinical context for patients, and how this may interact with or be impacted by PH treatments, may benefit from explicit exploration in future studies in a clinical, controlled setting. A volunteer sample was used for this research; therefore, self‐selection bias may be present. For example, individuals who struggle with their sleep may have been more inclined to take part. Although the prevalence of sleep difficulties in this sample is consistent with previous findings, future research may look to explore sleep difficulties using more robust methodology, including recruitment methods, as well as alternative approaches to examining sleep such as using objective methods. Ideally, this would include a longitudinal data collection period to increase confidence in a particular direction of effect.

## 5 Conclusion

Findings add to current literature revealing the high rates of sleep difficulties in people with PH. More specifically, the most frequent complaint was overall poor sleep quality, stemming particularly from issues with sleep disturbance and sleep latency. This was followed by insomnia and daytime sleepiness—which often co‐occurred. Depression partially explained the relationship between sleep quality, insomnia, and daytime sleepiness and HRQoL. Findings add to calls for routine screening of sleep difficulties in this clinical group and, when identified, to consider exploring co‐occurring depression symptoms given their statistical association.

## Author Contributions

JLG was responsible for the conceptualisation of the study, obtaining ethical approval, data collection and analysis, and write‐up under the supervision of GHR. VC was involved in data analysis and contributed to writing the manuscript for publication. IA provided expertise on PH and contributed to writing the manuscript for publication. ART provided expertise on clinical health psychology and contributed to writing the manuscript for publication. GHR was the project lead and JLG's primary supervisor. He supported JLG with all project tasks and contributed to writing the manuscript for publication. All authors reviewed and approved the final version for publication. ChatGPT was used to assist with improving the grammar and structure of sentences. Authors take full responsibility for the content of the publication.

## Funding

The authors have not declared a specific grant for this research from any funding agency in the public, commercial or not‐for‐profit sectors.

## Ethics Statement

Ethical approval was obtained from the University of Sheffield, Psychology Department Ethics Committee (067112).

## Consent

All participants provided consent to take part in the current study.

## Conflicts of Interest

The authors declare no conflicts of interest.

## Guarantor

Dr. Gregg H. Rawlings is the guarantor of the research article.

## Supporting information




**Table S1:** Spearman’s Rank Correlations for Continuous Variables and Outcome Measures, Pairwise deletion of missing data and inclusion of ‘PH Functional Class’ variable.
**Table S2:** Content analysis tables for PSQI qualitative additional sleep disturbance reasons; Themes of other reasons for sleep disturbance (Question 5j in PSQI).
**Table S3:** Themes of additional signs of restlessness seen by bed partner (Question 10e in PSQI).

## Data Availability

The data that support the findings of this study are available on request from the corresponding author. The data are not publicly available due to privacy or ethical restrictions.
