# Supplementary material for: How Do Sleep Difficulties Interact With Anxiety, Depression and Health‐Related Quality of Life in Pulmonary Hypertension?
Source: Clin Respir J. 2026 Jul 31;20(8):e70220. doi: 10.1111/crj.70220 (PMC13428227; doi:10.1111/crj.70220)
Supplement: Supplementary file 1 — Table S1: Spearman’s Rank Correlations for Continuous Variables and Outcome Measures, Pairwise deletion of missing data and inclusion of ‘PH Functional Class’ variable. Table S2: Content analysis tables for PSQI qualitative additional sleep disturbance reasons; Themes of other reasons for sleep disturbance (Question 5j in PSQI). Table S3: Themes of additional signs of restlessness seen by bed partner (Question 10e in PSQI). [file CRJ-20-e70220-s001.docx]

**Supplementary materials**

**Table 1 Spearman’s Rank Correlations for Continuous Variables and Outcome Measures, Pairwise deletion of missing data and inclusion of ‘PH Functional Class’ variable**

| Variable | 1 | 2 | 3 | 4 | 5 | 6 | 7 | 8 | 9 |
| --- | --- | --- | --- | --- | --- | --- | --- | --- | --- |
| 1. Age | - | - | - | - | - | - | - | - | - |
| 1. Length of Diagnosis | -.16  *n* = 111 | - | - | - | - | - | - | - | - |
| 1. PH Functional Class | .19  N = 53 | .02  N = 53 | - | - | - | - | - | - | - |
| 1. ESS (Daytime Sleepiness) | -.21*  N= 110 | .13  N = 110 | .22  N = 52 | - | - | - | - | - | - |
| 1. PSQI (Overall Sleep Quality) | .02  N = 104 | .30  N = 104 | .41**  N = 52 | .29**  N = 103 | - | - | - | - | - |
| 1. ISI (Insomnia) | -.02  N = 103 | .04  N = 103 | .32*  N = 53 | .42**  N = 102 | .71**  N = 99 | - | - | - | - |
| 1. emPHasis-10 (HRQoL) | -.04  N = 81 | -.16  N = 81 | .29*  N = 46 | .48**  N = 81 | .32**  N = 79 | .52**  N = 81 | - | - | - |
| 1. PHQ-9 (Depression) | -.12  N = 94 | -.11  N = 94 | .33*  N = 51 | .46**  N = 93 | .50**  N = 91 | .64**  N = 94 | .78**  N = 80 | - | - |
| 1. GAD-7 (Anxiety) | -.26*  N = 93 | .02  N = 93 | .28*  N = 51 | .35**  N = 92 | .31**  N = 90 | .49**  N = 93 | .66**  N = 80 | .75**  N = 92 | - |

Abbreviation: HRQoL, Health-Related Quality of Life

**p* < .05. ***p* < .01

**Table 2 Content analysis tables for PSQI qualitative additional sleep disturbance reasons; Themes of other reasons for sleep disturbance (Question 5j in PSQI)**

| Theme | Explanation of theme | Frequency (n) | Percentage (%) |
| --- | --- | --- | --- |
| Additional Health Conditions | Health conditions aside from PH, for example, arthritis, Crohn’s disease and the flu. | 10 | 24.3% |
| Anxiety | Comments about worrying and overthinking, for example, work anxiety and anxiety about dying. | 8 | 19.5% |
| Physical Discomfort | Sleep disturbance relating to physical factors such as pain, breathlessness and unable to get comfortable. | 8 | 19.5% |
| Struggling to sleep | Comments about difficulty getting to sleep. | 8 | 19.5% |
| General Disruption | Audible disruptions such as noise from traffic or bed partner moving. | 3 | 7.3% |
| Sleep Apnea | Sleep disturbance relating to Sleep Apnea. | 2 | 4.8% |
| Change of routine | Comment about difficulty sleeping after staying in hospital. | 1 | 2.4% |
| Stress | Comments about emotional stress and burn-out. | 1 | 2.4% |

*N = 41*

**Table 3 Themes of additional signs of restlessness seen by bed partner (Question 10e in PSQI)**

| Theme | Explanation of theme | Frequency (n) | Percentage (%) |  |
| --- | --- | --- | --- | --- |
| Turning Over | Comments relating to tossing and turning whilst sleep, or whilst trying to sleep. | 7 | 30.4% |  |
| Physical signs | | Physical discomfort, such as sneezing, leg pain and twitching. | 5 | 21.7% |
| Breathing Difficulties | Comments relating to breathing, for example difficulties with nose cannula and oxygen. | 3 | 13.0% |  |
|  |  |  |  |  |
| CPAP machine difficulties | Continuous Positive Airway Pressure machines are used as treatment for Sleep Apnea and involve a mask over the nose and mouth. | 3 | 13.0% |  |
| Disturbed by Partner | Comments about the bed partner moving in the night or leaving the room. | 3 | 13.0% |  |
| Talking in Sleep | Comments relating to humming or talking in sleep. | 2 | 8.7% |  |

Abbreviation: CPAP, Continuous Positive Airway Pressure machine

*N = 23*
